# Phosphorothioate DNA Mediated Sequence-Insensitive Etching and Ripening of Silver Nanoparticles

**DOI:** 10.3389/fchem.2019.00198

**Published:** 2019-04-16

**Authors:** Shengqiang Hu, Po-Jung Jimmy Huang, Jianxiu Wang, Juewen Liu

**Affiliations:** ^1^College of Chemistry and Chemical Engineering, Central South University, Changsha, China; ^2^Department of Chemistry, Waterloo Institute for Nanotechnology, University of Waterloo, Waterloo, ON, Canada

**Keywords:** oligonucleotides, phosphorothioate, silver nanoparticles, plasmonics, biosensors

## Abstract

Many DNA-functionalized nanomaterials and biosensors have been reported, but most have ignored the influence of DNA on the stability of nanoparticles. We observed that cytosine-rich DNA oligonucleotides can etch silver nanoparticles (AgNPs). In this work, we showed that phosphorothioate (PS)-modified DNA (PS-DNA) can etch AgNPs independently of DNA sequence, suggesting that the thio-modifications are playing the major role in etching. Compared to unmodified DNA (e.g., poly-cytosine DNA), the concentration of required PS DNA decreases sharply, and the reaction rate increases. Furthermore, etching by PS-DNA occurs quite independent of pH, which is also different from unmodified DNA. The PS-DNA mediated etching could also be controlled well by varying DNA length and conformation, and the number and location of PS modifications. With a higher activity of PS-DNA, the process of etching, ripening, and further etching was taken place sequentially. The etching ability is inhibited by forming duplex DNA and thus etching can be used to measure the concentration of complementary DNA.

## Introduction

Interfacing DNA with nanomaterials has resulted in many interesting hybrids for analytical (Liu and Lu, 2006; Liu et al., 2009; Zhou et al., 2017), nanotechnology (Wilner and Willner, 2012; Pu et al., 2014; Tan et al., 2014; Seeman and Sleiman, 2017; Shen C. et al., 2017; Chidchob and Sleiman, 2018; Hu et al., 2018), and biomedical applications (Qu et al., 2000; Cao et al., 2002; Liu et al., 2015; Lu et al., 2017; Sun et al., 2018). Such applied research in turn stimulated fundamental surface and biointerface studies (Herne and Tarlov, 1997; Storhoff et al., 2002; Liu, 2012; Carnerero et al., 2017). Most of previous research focused on DNA-directed assembly (Mirkin et al., 1996; Liu and Lu, 2003; Sharma et al., 2009; Chou et al., 2014; Liu and Liu, 2017; Lin et al., 2018), or DNA-templated growth of nanomaterials (Nykypanchuk et al., 2008; Surwade et al., 2013; Wu et al., 2014; Song et al., 2015), while etching or dissolution of nanoparticles by DNA was much less explored. We reason that such studies are also important for the following reasons. First, nanoparticles were always assumed to be stable during DNA conjugation or assembly. If DNA can dissolve nanoparticles, such assumptions need to be updated and care has to be taken for long-term storage of such materials. In addition, DNA-mediated etching of nanoparticles can be a way of controlled release. Finally, it can further our fundamental understanding of DNA/nanoparticle interfaces.

Using a relatively high concentration of DNA (e.g., >1 μM), we recently observed etching of silver nanoparticles (AgNPs) by DNA oligonucleotides (Hu et al., 2019). For spherical AgNPs, poly-cytosine (poly-C) was the most effective, while the other three types of homopolymers did not display an obvious effect. The base composition of DNA is critical for etching silver-based nanomaterials.

**Figure 1:**
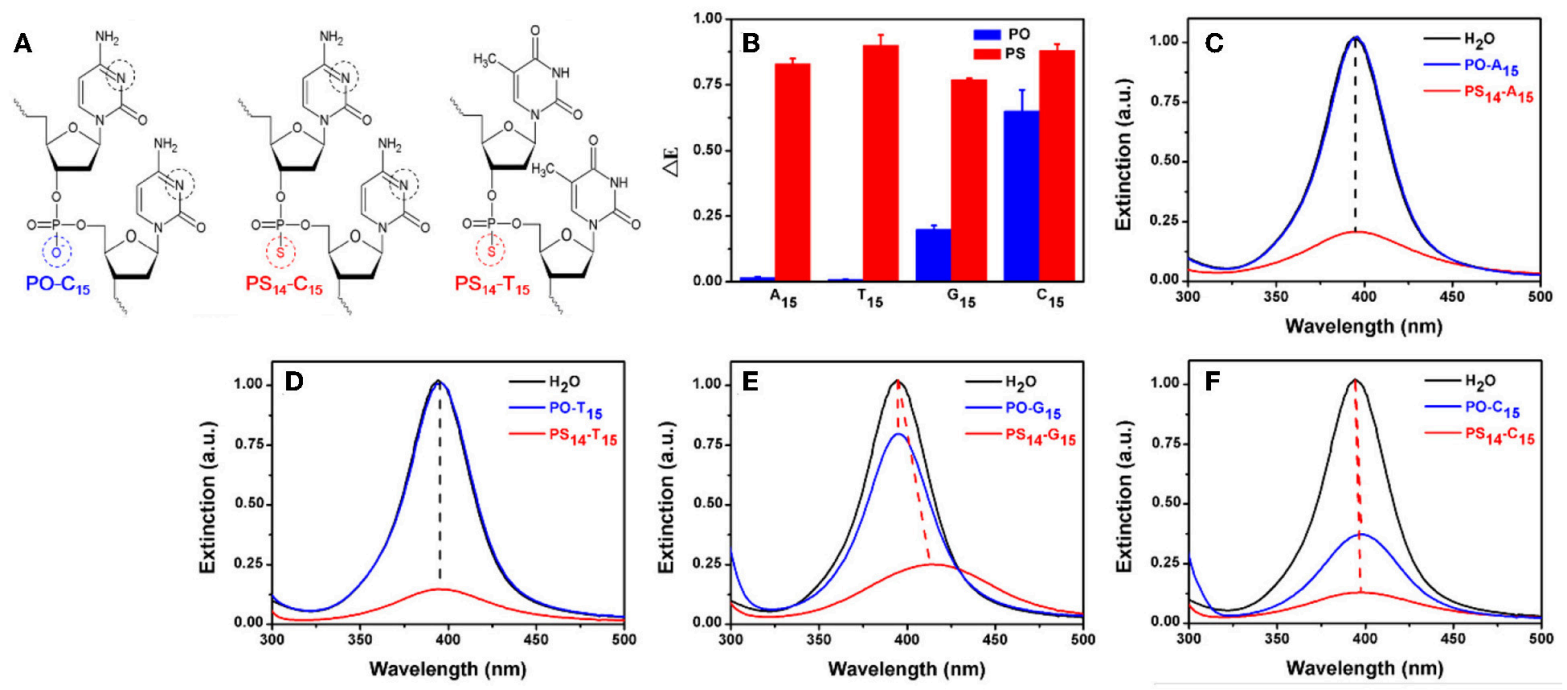
**(A)** The structure of PO- and PS-DNA linkages for poly-C and poly-T DNA. The potential binding sites of DNA to AgNPs are marked in black and red circles. **(B)** The dropped extinction at 395 nm induced by 10 μM of 15-mer PO-DNA and PS-DNA. UV-vis spectra of the AgNPs treated with PO- and PS-DNA with the sequences of **(C)** A_15_, **(D)** T_15_, **(E)** G_15_, and **(F)** C_15_. The red dash lines represent red shifted spectra, while the black lines are for non-shifted spectra.

Poly-C DNA can effectively etch AgNPs, but the required high DNA concentration and specific DNA sequence restricted its applications in analytical detection and controlled release. So far, we have studied only unmodified DNA. We reason that the effect of DNA might be further improved by introducing modifications with stronger metal ligands. Phosphorothioate (PS) modification refers to replacing one of the non-bridging oxygen atoms by sulfur (Figure 1A) (Huang and Liu, 2014, 2015; Huang et al., 2015a,b; Liu et al., 2018). The PS sites on DNA can bind strongly to thiophilic metals (e.g., Au and Ag) and PS-modified DNA (PS-DNA) has been used for nanomaterial synthesis (Ma et al., 2008; Farlow et al., 2013; Weadick and Liu, 2015; Shen J. et al., 2017), nanostructure assembly (Jiang et al., 2005; Lee et al., 2007; Pal et al., 2009; Shen J. et al., 2017), and biosensing (Zhang et al., 2009; Huang P. J. J. et al., 2016). We previously compared adsorption of PS-DNA with normal phosphodiester DNA (PO-DNA) on AuNPs, and concluded that the PS-DNA was more strongly adsorbed (Zhou et al., 2014). PS-DNA was also used to functionalize quantum dots (Ma et al., 2008; Farlow et al., 2013). In addition, PS modifications have been used to probe the reaction mechanism of ribozymes (Cunningham et al., 1998; Huang and Liu, 2014; Huang et al., 2015a, 2019; Thaplyal et al., 2015). All these studies took advantage of the strong affinity between PS and thiophilic metals. Since silver is also strongly thiophilic, we speculate that PS-DNA may be more effective for etching AgNPs in a less DNA sequence-dependent manner.

In this work, we systematically studied the effect of PS modifications and found that it could significantly decrease the needed DNA concentration. At the same time, the sequence of DNA was less important, making DNA-mediated etching available for many more sequences. The effects of pH, DNA length, the number and location of PS modifications and DNA conformation were also systematically studied and compared with the normal DNA of the same sequences, leading to interesting multi-stage etching and ripening process and chemically controlled etching.

## Materials and Methods

### Chemicals

All the DNA were purchased from Integrated DNA Technologies (IDT, Coralville, IA, USA), and their sequences are shown in Table S1. Commercial citrate-capped AgNPs (20 nm diameter) were purchased from Nanocomposix (San Diego, CA, USA). Trisodium citrate and 3-(N-morpholino)propanesulfonic acid (MOPS) were obtained from Sigma-Aldrich (St. Louis, MO). Sodium nitrate (NaNO_3_) was purchased from Mandel Scientific (Guelph, ON, Canada).

### Instrumentation

UV-vis absorption spectra were recorded on a spectrometer (Agilent 8453A). The morphology of AgNPs was examined by a transmission electron microscope (TEM, Philips CM10). The etching kinetics of AgNPs were monitored using a microplate reader (SpectraMax M3). Dynamic light scattering (DLS) measurements were carried out using a Zetasizer Nano 90 (Malvern) at 25°C. Circular dichroism (CD) spectra were collected on a Jasco J-715 spectrophotometer (Jasco, Japan).

### Comparison of Etching by PO and PS-DNA

In a typical experiment, a 15-mer DNA (20 μM, 35 μL) was incubated with an equal volume of AgNPs (10 μg/mL) at 37°C for 1.5 h. The final concentration of DNA was 10 μM. Then, the sample was analyzed by a spectrometer.

### Effect of pH on Etching Kinetics

Typically, PO-C_15_, PS_14_-C_15_, or PS_14_-T_15_ (20 μM, 50 μL) was mixed with the AgNPs (10 μg/mL, 50 μL) in a 96-well plate. Then, 10 μL of 10 mM buffer with different pH values (citrate buffer for 4.0, 5.0, and 6.0; MOPS for 7.0 and 7.9) was added and incubated at 37°C for 1.5 h. The absorbance intensity was monitored at 395 nm every 0.5 min under the kinetic mode using the plate reader.

### PS-DNA Structure Dependent Etching

First, PS-R DNA (40 μM, 17.5 μL) was mixed with 17.5 μL of its cDNA of different concentrations (5.0, 10, 20, 40, 60, 80, and 100 μM), followed by the addition of NaNO_3_ (0.25 M, 2.0 μL). Then, the mixture was heated to 95°C for 5 min and cooled slowly to room temperature. Finally, AgNPs (10 μg/mL, 35 μL) were added and incubated at 37°C for 1.5 h. The final concentrations of the cDNA were 1.25, 2.5, 5.0, 10, 15, 20, and 25 μM, respectively.

## Results and Discussion

### PS-DNA Mediated Etching of AgNPs

**Figure 2:**
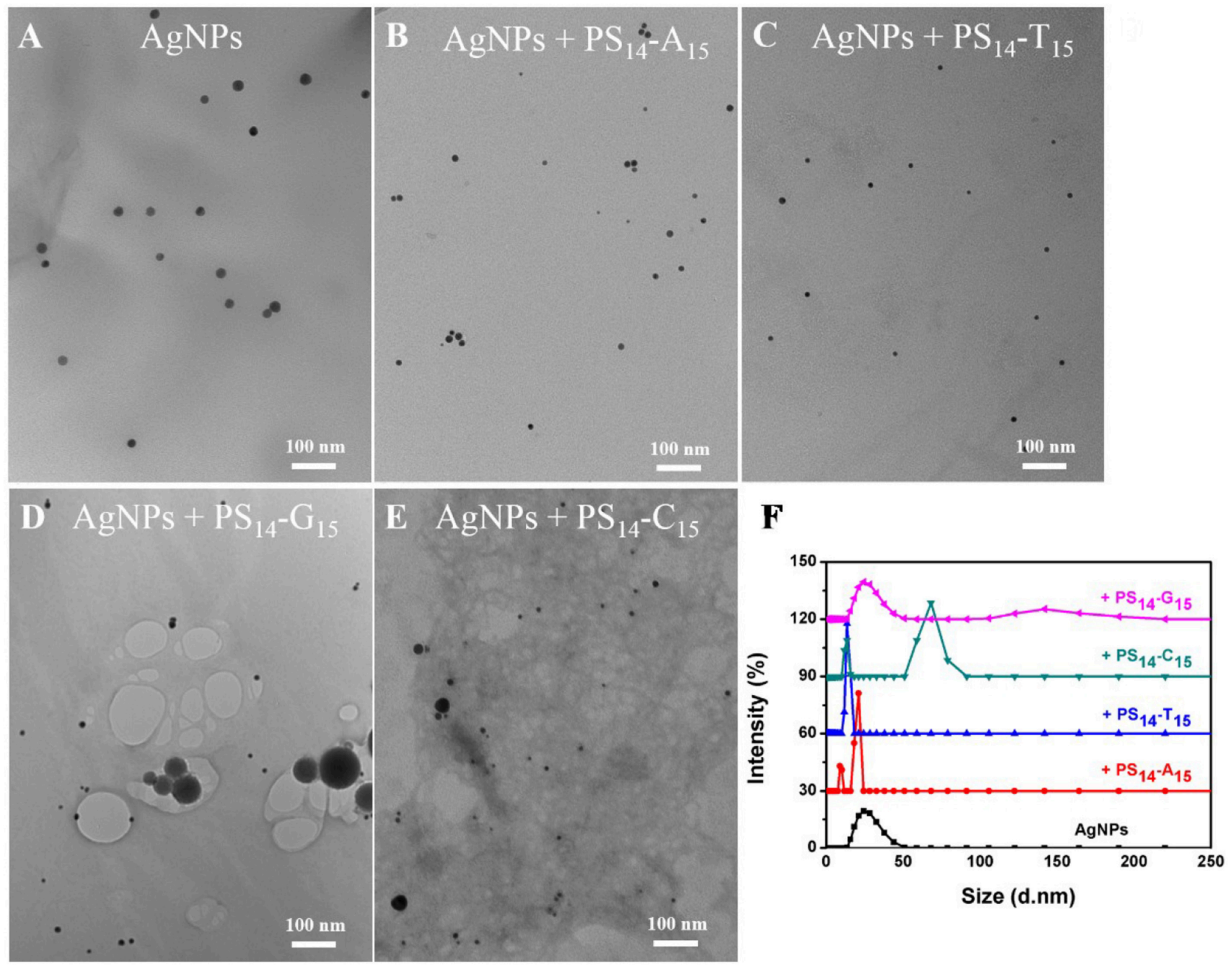
TEM micrographs of **(A)** untreated 20 nm AgNPs, and the AgNPs treated with 10 μM **(B)** PS_14_-A_15_, **(C)** PS_14_-T_15_, **(D)** PS_14_-G_15_, and **(E)** PS_14_-C_15_. **(F)** The average hydrodynamic diameter of the AgNPs etched by 10 μM of various 15-mer PS-DNA.

To test the effect of PS modification (see Figure 1A for its structure), we used the four types of 15-mer DNA homopolymers both with the normal phosphodiester (PO) backbone and with full PS modifications (each bridging phosphate contained a PS modification). Our 20 nm AgNPs had a strong surface plasmon peak at 395 nm (Figure 1C). Adding the normal PO-A_15_ DNA had no effect and the UV-vis spectrum retained its original shape. In contrast, the PS_14_-A_15_ (note that a 15-mer DNA has only 14 bridging phosphates) dropped the extinction peak intensity by over 80%. From this experiment, we concluded that the sulfur atoms in the PS-DNA were the reason for the decreased extinction of the AgNPs. From TEM (Figure 2A and Figures S1, S2A), our starting AgNPs were monodispersed ~20 nm spheres. After adding the PS_14_-A_15_, overall the AgNPs became smaller (Figures 2B,F), indicating its etching.

Similar experiments were also performed with the other DNA sequences, and the same observations were also made with the two T_15_ DNAs from both their UV-vis spectra (Figure 1D) and TEM (Figure 2C). The lack of etching by PO-A_15_ and PO-T_15_ is in agreement with the relatively low affinity between these two DNA bases and silver surface (Basu et al., 2008; Wu et al., 2014).

When the PO-G_15_ was added, the extinction intensity of the AgNPs dropped by about 20% (Figure 1E), while a nearly 80% drop was observed when PS_14_-G_15_ was added. At the same time, the peak red shifted by 18 nm. This suggested formation of larger AgNPs, which was confirmed by TEM (Figure 2D). Therefore, with this DNA concentration and reaction time, the PS_14_-G_15_ helped Ostwald ripening of the AgNPs. Etching was the first step of the interaction, where the AgNPs were dissolved by the added DNA. Extensive etching and deposition of dissolved silver species on larger AgNPs (thus with lower solubility) resulted in the subsequent ripening.

The PO-C_15_ DNA was very effective in etching the AgNPs and it decreased extinction by 65% (Figure 1F). The extinction of the PS_14_-C_15_-treated sample further decreased the extinction peak to nearly 90%. Interestingly, under this condition, the ripening process caused only a 3 nm redshift, which was much smaller than that induced by PS_14_-G_15_ (18 nm). Meanwhile, the average size of the PS_14_-C_15_-treated AgNPs (Figure 2E) was smaller than that of the PS_14_-G_15_ treated sample (Figure 2D), indicating the cytosine and guanine bases also played a role on etching.

With these four pairs of DNA, we plotted the peak intensity drop in Figure 1B. All the PS sequences dropped the intensity by a similar value (the red bars), while a much larger difference was observed with the normal PO-DNA (blue bars). Since all the PS-DNA sequences had a significant etching effect, PS-DNA can etch the AgNPs in a less sequence-independent manner. This might be useful since etching can be general to different DNA sequences.

### Comparison of PS and DNA Base Coordination

The above experiments indicated that PO-T_15_ is one of the least effective sequences for etching AgNPs. Therefore, the etching effect of PS_14_-T_15_ should mainly come from the PS sites. PO-C_15_ is the best PO DNA and only the bases are available for etching, while PS_14_-C_15_ is likely to be a most effective sequence overall. For PS_14_-C_15_, both the PS sites and the cytosine bases are likely to contribute to silver binding. Since these three sequences are representative, they were chosen for further studies. Their silver coordination sites are marked in black and red circles in Figure 1A.

**Figure 3:**
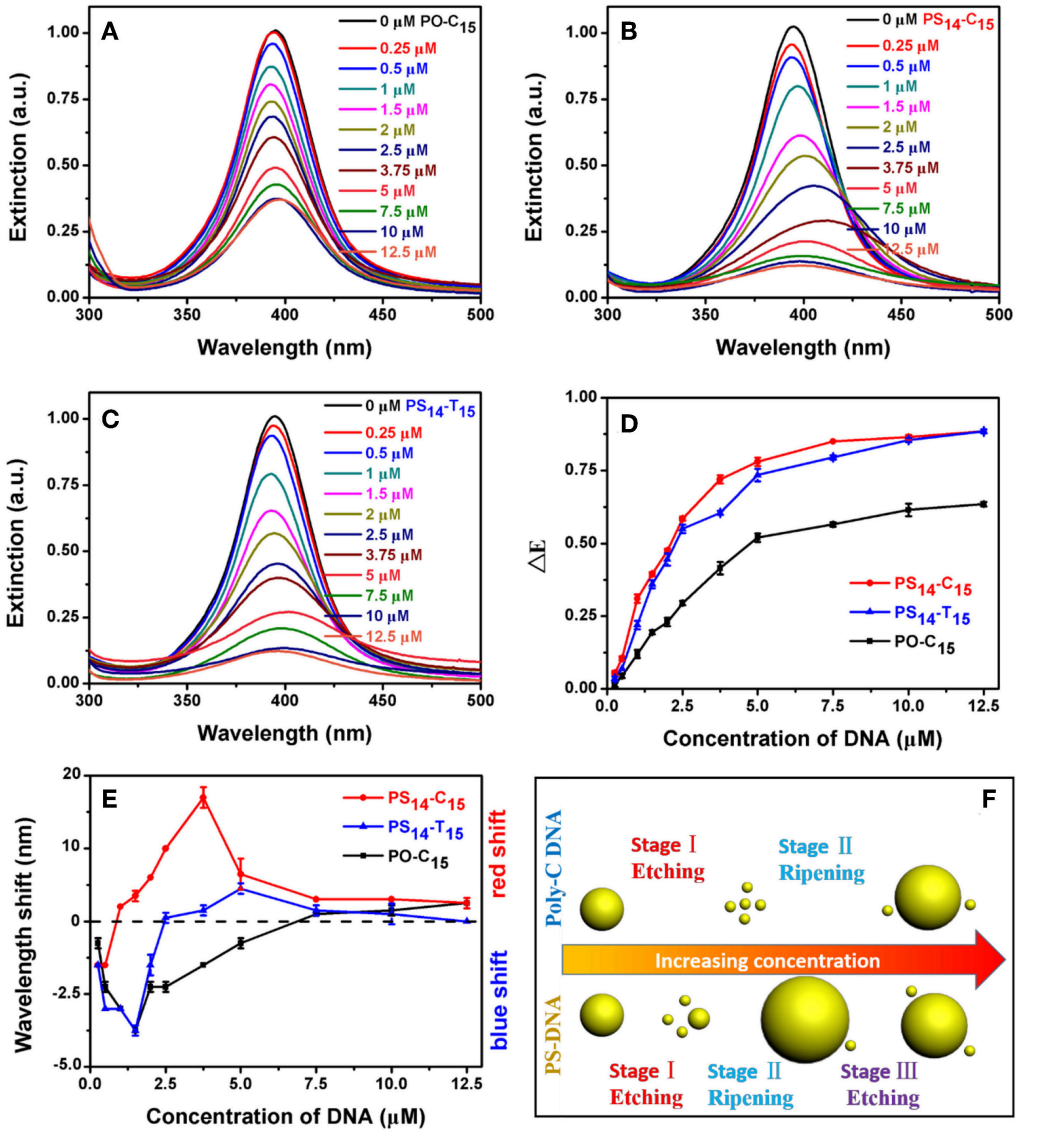
**(A)** PO-C_15_, **(B)** PS_14_-C_15_, and **(C)** PS_14_-T_15_ concentration-dependent etching of the AgNPs. Relationship between DNA concentration and **(D)** dropped extinction and **(E)** wavelength shift of the AgNPs. **(F)** Comparison of the AgNPs etched by poly-C PO and PS DNA. With a stronger silver binding affinity, the PS DNA achieved the stage III reaction of further etching the larger AgNPs produced from the previous ripening stage.

**Figure 4:**
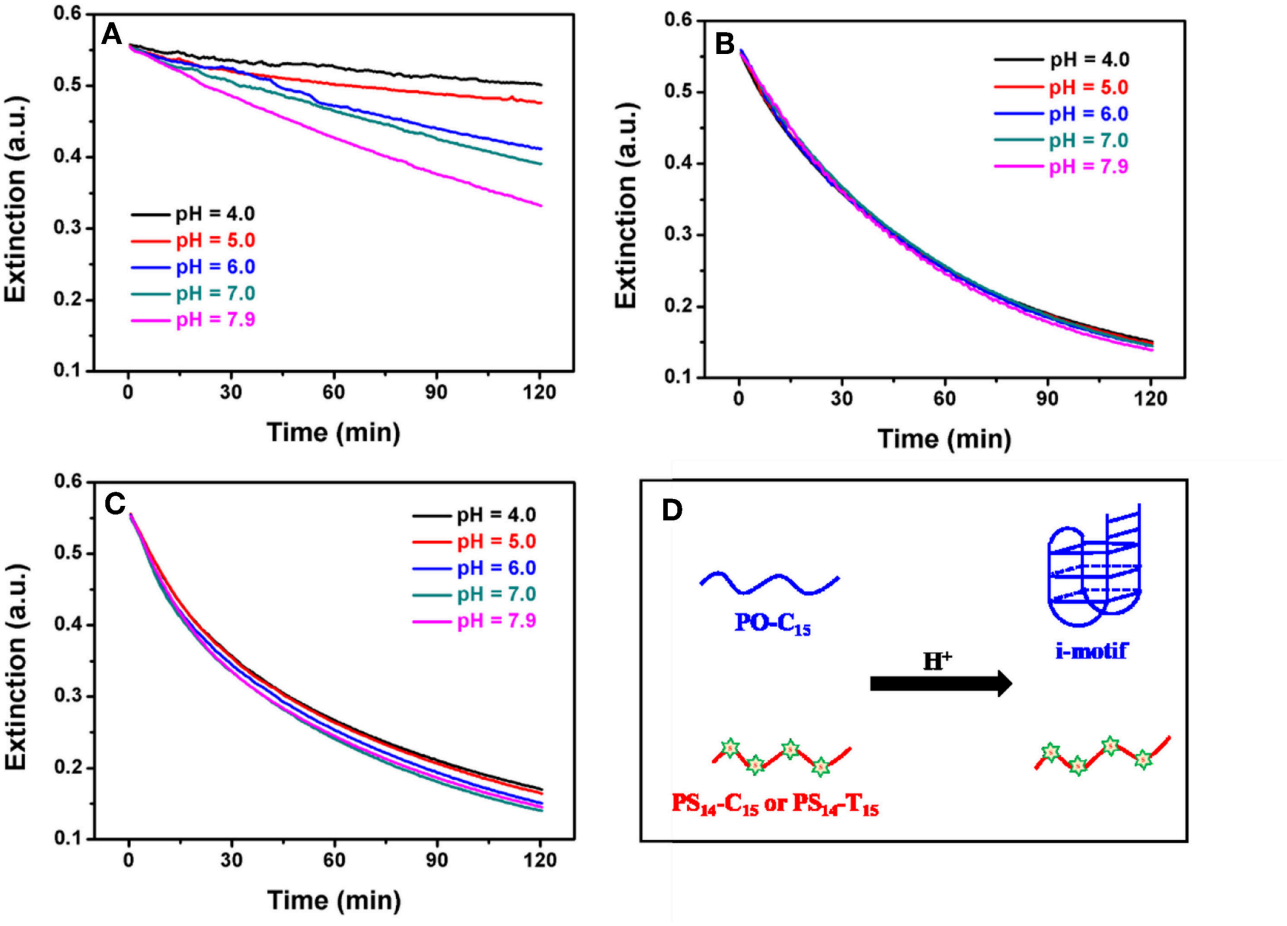
Effect of pH on the kinetics of **(A)** PO-C_15_, **(B)** PS_14_-C_15_, and **(C)** PS_14_-T_15_ mediated AgNPs etching. Citrate buffer was used for pH 4.0, 5.0, and 6.0, and MOPS buffer was used for pH 7.0 and 7.9. **(D)** Effect of low pH on the structure of some PO and PS DNA.

We first studied the effect of DNA concentration. In each case, the peak intensity decreased with increase of DNA concentration (Figures 3A–C). At the same time, some redshifted peaks were observed at high DNA concentrations. All these experiments were performed with an incubation time of 1.5 h, when the systems were approaching equilibrium (Figure 4).

By plotting the decrease of peak height against DNA concentration, we obtained their apparent binding curves (Figure 3D). Among these DNAs, PO-C_15_ had the lowest response with an apparent *K*_d_ of 3.87 μM. PS_14_-C_15_ had a tighter binding with a *K*_d_ of 1.78 μM. Interestingly, PS_14_-T_15_ had a *K*_d_ similar to that of PS_14_-C_15_, and thus from this standpoint, the base's contribution was minimal. Even at a relatively low DNA concentration, the base did not contribute much to etching. Adding PS modifications decreased the concentration requirement for C_15_ by 2.2-fold, while the improvement for T_15_ was close to infinity (compared to PO-T_15_).

We then plotted the shift of peak wavelength (Figure 3E), where the upper half of the figure is for red shifted samples and the lower half for blue shifts. With low concentrations of PO-C_15_, a gradual blue-shift of the AgNPs peak was observed and the maximal shift was achieved with 1.5 μM DNA, indicating etching of the AgNPs to form small particles. Then, the peak started to red shift attributable to Ostwald ripening. When the DNA concentration was more than 7.5 μM (crossing the dashed line in Figure 3E), the peak red-shifted relative to the original AgNPs.

For PS_14_-C_15_, most of their spectra were red shifted (e.g., ripening) except for the DNA concentration below 1 μM. From TEM, the overall size was indeed decreased for the 0.5 μM PS_14_-C_15_ treated sample (Figure S2B). A wide size distribution with both larger and smaller AgNPs was observed for more PS_14_-C_15_ (7.5 μM), confirming the ripening (Figure S2C). However, we cannot rule out slight aggregation of AgNPs occurring at the same time, which also caused the red shifted spectra. By comparing PO-C_15_ and PS_14_-C_15_, both showed etching at low DNA concentrations and then ripening with more DNA added. PS_14_-C_15_ has a tighter affinity with silver allowing it to achieve the etching-to-ripening transition at a lower DNA concentration.

Interestingly, for PS_14_-C_15_, the red shift initially increased but later decreased when the concentration of PS_14_-C_15_ was more than 3.75 μM (red trace in Figure 3E). A similar trend was also observed for PS_14_-T_15_ despite smaller shifts compared to that of PS_14_-C_15_. This difference may be ascribed to their different DNA bases, suggesting that the cytosine bases of PS_14_-C_15_ also participated in the etching process. We reason that PS_14_-C_15_ had a complex multi-stage etching process. Low concentration of DNA contributed to AgNPs etching and further ripening (Figures S2A–C). Further increased PS_14_-C_15_ DNA could further etch the larger AgNPs from the previous ripening step, which yielded the decreased red shift. The etching and thus size decrease was also confirmed by TEM (Figure S2D). As a result, we proposed a three-stage mechanism for PS-DNA to interact with AgNPs: etching, ripening and further etching (Figure 3F). For the PO-DNA, we only observed two stages (etching-ripening of AgNPs) indicating that cytosine bases alone were incapable of etching larger AgNPs, which were thermodynamically more stable than the originally used 20 nm ones. Since PS_14_-T_15_ was also not very obvious than PS_14_-C_15_ for this three-stage process, both cytosine bases and PS of PS_14_-C_15_ contribute to the etching-to-ripening transition (with PS being the major contributor).

### Kinetics and Effect of pH

To further study etching, we followed the reaction kinetics. Since the conformation of poly-C DNA is strongly affected by pH (Dong et al., 2014; Huang Z. et al., 2016), we also measured the etching kinetics at different pH values. For PO-C_15_, etching was strongly inhibited at low pH (Figure 4A). In particular, when pH was at 6 or lower, etching was essentially fully inhibited. In contrast, PS_14_-C_15_ had the same rate of etching regardless of pH from 4 to 7.9 (Figure 4B). We fitted the kinetic data of the PS-DNA to a first-order equation and obtained a rate constant of 41.3 h^−1^, which was much faster than the PO kinetics of 183.4 h^−1^ at pH 7.9 (the rate of the PO samples was even slower at lower pH). Since the only difference here was the base, we reason that the inhibited PO-C_15_ etching must be related to its base protonation and formation of secondary structures such as the i-motif (Figure 4D).

Using circular dichroic (CD) spectroscopy, a strong positive peak at around 285 nm and a small negative peak near 260 nm were observed suggesting an intramolecular i-motif structure of PO-C_15_ at pH 4.0 (the black spectrum in Figure S3) (Liu and Balasubramanian, 2003). Such a folded conformation could shield the bases and inhibit their interaction with AgNPs or with Ag^+^. For the PS_14_-C_15_-mediated etching, pH had no effect on etching. Since the PS modifications could cause a reduced melting temperature compared to the PO counterpart (Gonzalez et al., 1991), the PS_14_-C_15_ was incapable of forming i-motif at 37°C even under acidic conditions (the red spectrum in Figure S3). Therefore, the exposed PS and the bases in the random-coil structured PS_14_-C_15_ could serve as the ligand for etching the AgNPs. The pH-independent etching also appeared for PS_14_-T_15_ (Figure 4C), demonstrating the generality of PS-DNA-mediated etching of AgNPs.

### The Number of PS Modifications and AgNP Etching

**Figure 5:**
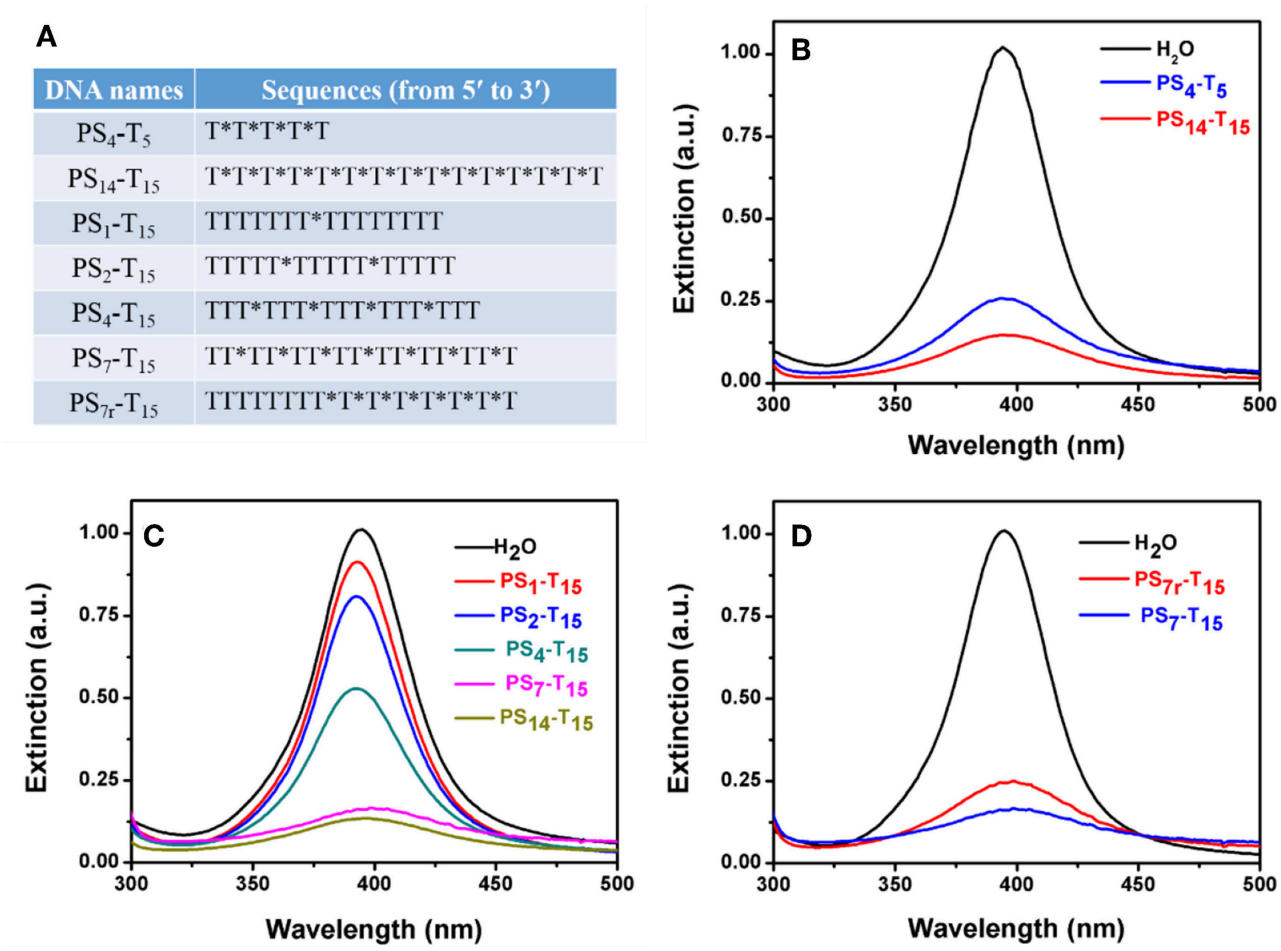
**(A)** The PS-DNA sequences used for studying the number of PS modifications. PS-DNA mediated AgNP etching as a function of **(B)** poly-T DNA length, **(C)** the number, and **(D)** the location of PS modifications. The final concentration of PS_4_-T_5_ was 35 μM, while the concentration of all other PS-DNAs was 10 μM.

The above experiments used 15-mer DNA with full PS modification. We then varied the number of DNA length and PS modifications (see Figure 5A for the DNA sequences). First, the DNA length was explored. To minimize the effect of the DNA base, PS-modified poly-T DNAs were tested. The length of DNA varied from 5-mer to 15-mer, and the total PS modification was maintained to be the same (e.g., the molar concentration of PS_4_-T_5_ was 3.5 times of that of PS_14_-T_15_). The peak of the PS_14_-T_15_ sample dropped more than that of PS_4_-T_5_, suggesting that longer DNA was more effective and thus the importance of polyvalent binding (Figure 5B).

We then varied to the number of PS modifications, while the DNA length was maintained at 15-mer. The number of PS modifications was reduced from 14 to 7, 4, 2, and 1 (Figure 5C). The peak intensity gradually dropped with increased PS modifications. For the poly-T DNAs with 1–7 PS modifications, the drop in the peak intensity was linearly proportional to the number of PS (Figure S4), further highlighting that the PS responsible for the AgNP etching. This also provided a method to quantitatively tune the extent of etching. Further increase of the PS modifications to 14 did not bring in much more changes, suggesting that seven PS modifications could be sufficient with the 1.5 h incubation time.

Finally, we explored the effect of the location of PS modifications. Compared to the uniform distribution of PS in the whole DNA backbone of PS_7_-T_15_, the 7 PS modifications in PS_7r_-T_15_ were concentrated on the 3′-terminus of the DNA. Interestingly, the evenly distributed PS_7_-T_15_ had a stronger decrease (Figure 5D), implying that PS coordination is more effective when they are separated.

### DNA Conformation Dependent Etching

**Figure 6:**
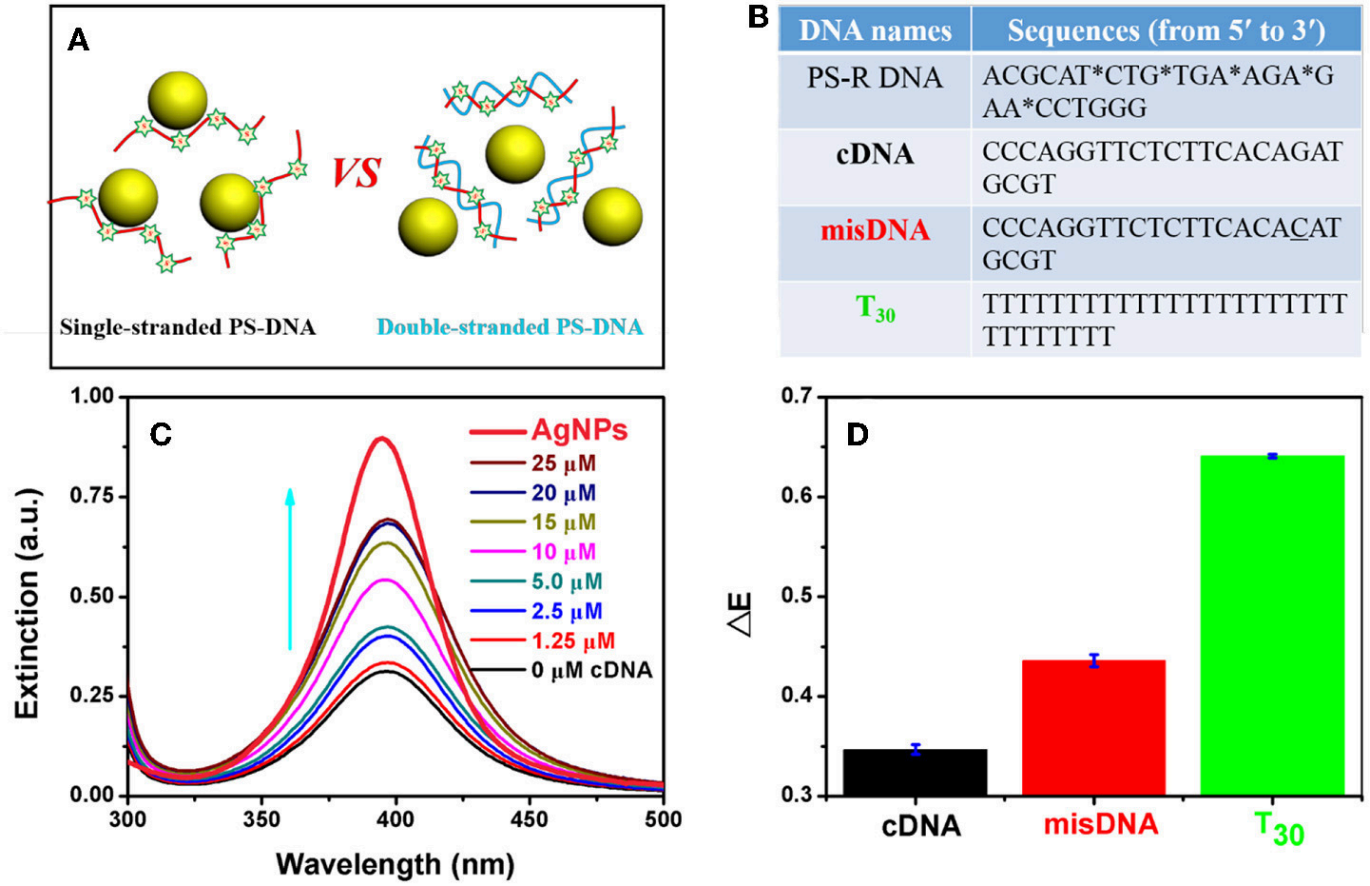
**(A)** Schematics and **(B)** DNA sequences used for PS-DNA conformation-dependent etching of AgNPs. The mistached base is underlined. **(C)** UV-vis spectra of the AgNPs mixed with the PS-R DNA in the presence of various concentrations of the cDNA. The spectrum of the AgNPs (no DNA) is in bold with red color. The arrow points increased cDNA concetnration. **(D)** Dropped extinction of the AgNPs with the PS-R DNA mixed with 25 μM of different DNA sequences.

Effective adsorption of PS-DNA on AgNPs could be important for the etching process. All the above experiments used flexible single-stranded DNA oligonucleotides, while a rigid DNA structure (e.g., duplex) may hinder the attachment of DNA to AgNPs due to restricted binding sites (Figure 6A). To test this hypothesis, we explored the effect of DNA conformation on etching by forming duplex DNA. However, PS modifications can weaken the stability of duplex DNA as reflected from the reduced melting temperature (*T*_m_) (Gonzalez et al., 1991). Furthermore, the A-T base pair with a PS modification showed more decreased *T*_m_ than that of the C-G base pair (Stein et al., 1988). Therefore, we designed a PS-modified random DNA (named PS-R DNA) with a high GC content (Figure 6B). This DNA could etch the AgNPs (the black spectrum in Figure 6C), and the etching efficiency was gradually inhibited with increasing dose of the complementary DNA (cDNA). The inhibiting efficacy was sharply decreased when the misDNA with a single mismatched base was added, while a full non-complementary DNA (T_30_) had little inhibition effect (Figure 6D). Therefore, we can attribute the cDNA-dependent etching to the formation of the duplex DNA. In other words, single-stranded DNA is much more effective for etching the AgNPs, although the PS backbone is still fully exposed in duplex DNA. This implies that DNA needs to fold into optimal binding structures, and etching cannot take place effectively on isolated PS sites.

## Conclusions

In summary, we reported that PS modifications on DNA could improve etching of AgNPs in several aspects. First, the sequence generality is significantly expanded, and the introduced PS allows essentially any DNA sequence to etch AgNPs beyond just poly-C DNA. Furthermore, the required DNA concentration decreased clearly, and at the same DNA concentration the rate of etching was much faster than that with PS modifications. The etching process also effectively took place for PS-DNA despite the low pH, which could inhibit etching induced by normal PO-DNA (e.g., poly-C DNA). At the same time, we could control the etching efficacy through changing DNA length and the number and location of PS modifications. With stronger etching efficiency, the reaction process was found to contain three stages: etching by low concentrations of PS-DNA, followed by Ostwald ripening at medium DNA concentrations, and further etching in the presence of high DNA concentrations. This work has expanded the scope of the interaction between DNA and nanomaterials, and it might lead to interesting analytical and biomedical applications. For example, etching of various silver nanostructures may produce visible color change for colormetric biosensors. These sensors might detect multiple analytes by using aptamers and by designing strategies to target the PS sites. At the same time, it also calls for attention regarding the stability of nanomaterials when designing hybrid materials containing silver nanoparticles (and potentially other materials) with DNA.

## Author Contributions

SH, JW, and JL designed the experiments and wrote the paper. SH performed the experiments. PH contributed in the DNA design. All authors read and approved the final version of the manuscript.

### Conflict of Interest Statement

The authors declare that the research was conducted in the absence of any commercial or financial relationships that could be construed as a potential conflict of interest.
