# Supplementary material for: Phosphorothioate DNA Mediated Sequence-Insensitive Etching and Ripening of Silver Nanoparticles
Source: Front Chem. 2019 Apr 16;7:198. doi: 10.3389/fchem.2019.00198 (PMC6476897; doi:10.3389/fchem.2019.00198)
Supplement: Supplementary file 1 [file Data_Sheet_1.pdf]

Supporting Information

Phosphorothioate DNA mediated sequence-insensitive etching and ripening of silver nanoparticles

Shengqiang Hu,[1,2] Po-Jung Jimmy Huang,[2] Jianxiu Wang[1]* and Juewen Liu[2]*

[1] College of Chemistry and Chemical Engineering, Central South University, Changsha 410083, China *E-mail: jxiuwang@csu.edu.cn

[2] Department of Chemistry, Waterloo Institute for Nanotechnology, University of Waterloo, Waterloo, Ontario, N2L 3G1, Canada *E-mail: liujw@uwaterloo.ca

Table 1. The DNAs sequences and modifications used in this work. The PS modifications are denoted by the asterisks.

**DNA names**	**Sequences (from 5′ to 3′)**
**PO-A_{15}**	AAAAAAAAAAAAAAA
**PO-T_{15}**	TTTTTTTTTTTTTTT
**PO-C_{15}**	CCCCCCCCCCCCCCC
**PO-G_{15}**	GGGGGGGGGGGGGGG
**PS_{14}-A_{15}**	A*A*A*A*A*A*A*A*A*A*A*A*A*A*A
**PS_{14}-T_{15}**	T*T*T*T*T*T*T*T*T*T*T*T*T*T*T
**PS_{14}-C_{15}**	C*C*C*C*C*C*C*C*C*C*C*C*C*C*C
**PS_{14}-G_{15}**	G*G*G*G*G*G*G*G*G*G*G*G*G*G*G
**PS_{4}-T_{5}**	T*T*T* T*T
**PS_{1}-T_{15}**	TTTTTTT*TTTTTTTT
**PS_{2}-T_{15}**	TTTTT*TTTTT*TTTTT
**PS_{4}-T_{15}**	TTT*TTT*TTT*TTT*TTT
**PS_{7}-T_{15}**	TT*TT*TT*TT*TT*TT*TT*T
**PS_{7r}-T_{15}**	TTTTTTTT*T*T*T*T*T*T*T
**PS-R DNA**	ACGCAT*CTG*TGA*AGA*GAA*CCTGGG
**cDNA**	CCCAGGTTCTCTTCACAGATGCGT
**misDNA**	CCCAGGTTCTCTTCACACATGCGT
**T_{30}**	TTTTTTTTTTTTTTTTTTTTTTTTTTTTTT

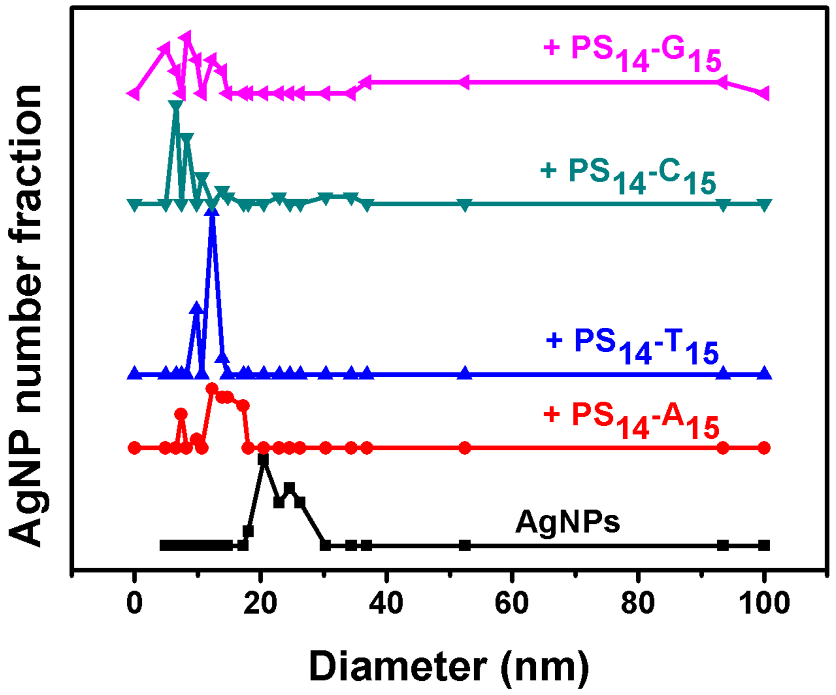

Figure S1. A histogram of the particle size distribution of AgNPs from the TEM data. The smaller and bigger size than 20 nm was due to etching and ripening of the AgNPs by the PS-DNAs, respectively.

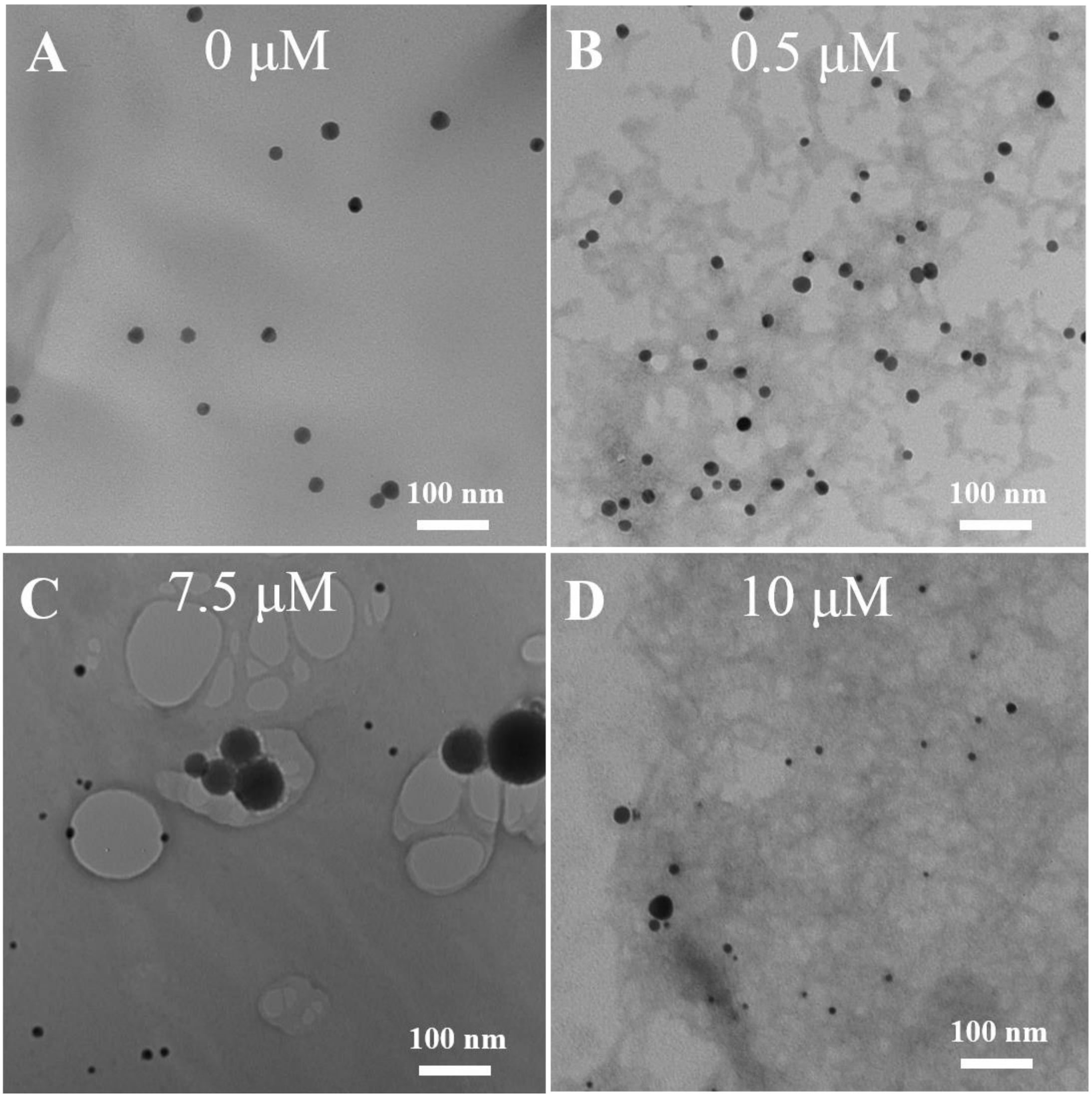

Figure S2. TEM micrographs of 5 μg/mL AgNPs in the absence (A) and presence of (B) 0.5 μM, (C) 7.5 μM and (D) 10 μM PS_{14}-C_{15}.

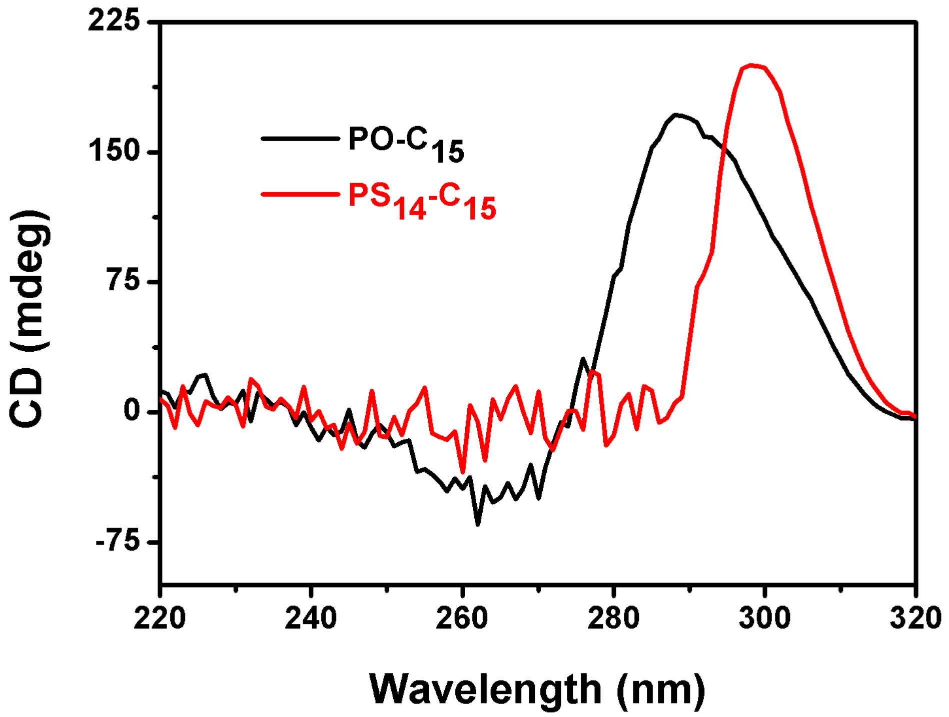

Figure S3. CD spectra of 10 μM PO-C_{15} and PS_{14}-C_{15} at pH 4.0 in citrate buffer. The positive peak at around 285 nm and the negative peak near 260 nm demonstrated i-motif structure of PO-C_{15}, while the PS DNA failed to form the i-motif under this condition.

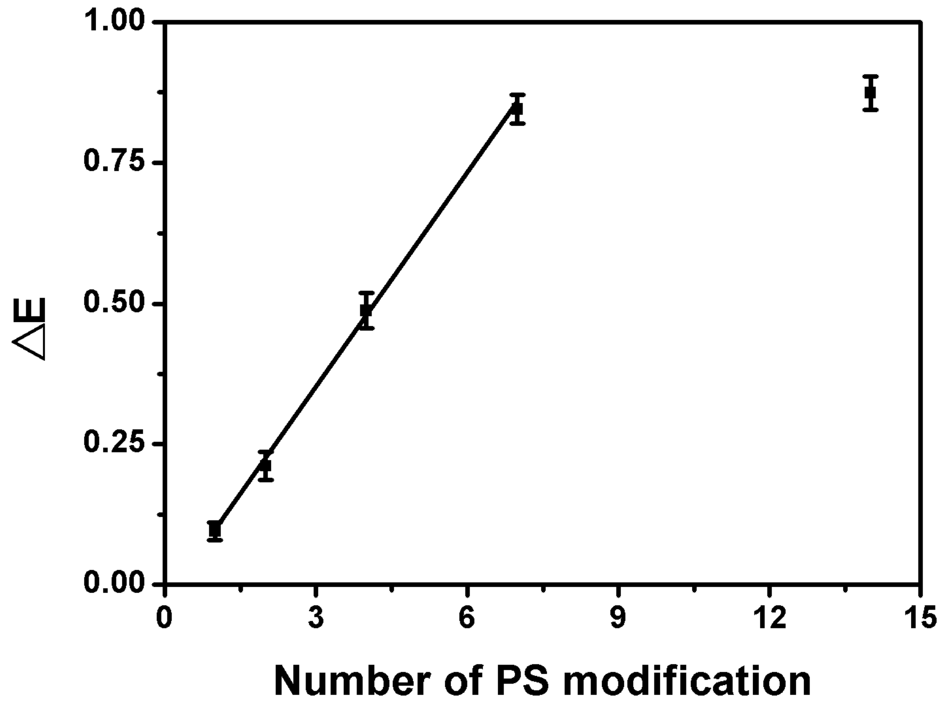

Figure S4. The dropped extinction as a function of the number of PS modifications. A linear relationship was obtained when the number of PS modifications ranged from 1, 2, 4 to 7.
